# Evaluation of urban community digital landscape and runoff regulation effect by GIS and InVEST model

**DOI:** 10.1371/journal.pone.0352335

**Published:** 2026-06-29

**Authors:** Lina Yan, Xin Gu

**Affiliations:** College of Artificial Intelligence Xi'an Siyuan University, Xi'an, China; Sathyambama Institute of Science and Technology: Sathyabama Institute of Science and Technology (Deemed to be University), INDIA

## Abstract

In high-density urban communities, the expansion of impervious surfaces and the fragmentation of green spaces increase surface runoff. Accurate identification of runoff regulation effects at the community scale has therefore become an important issue in landscape optimization. This study examined a typical high-density urban community. A Geographic Information System (GIS) was used to process high-resolution remote sensing images, terrain data, and meteorological records. An eight-class digital landscape classification system was then established. The Integrated Valuation of Ecosystem Services and Trade-offs–Sediment Delivery Ratio (InVEST-SDR) module was combined with landscape pattern metrics to construct a community-scale runoff regulation assessment framework. Model parameters were calibrated using observed runoff data. Runoff regulation results under current conditions and optimized scenarios were subsequently compared. The results showed that the model achieved a coefficient of determination of 0.89 and an average relative error of 1.3%, indicating reliable performance at the community scale. When impervious surfaces accounted for 37.2% of the area, the mean annual runoff depth reached 41.8 mm and the runoff coefficient was 0.52. After increasing green-space coverage by 15% and patch aggregation by 23%, the mean annual runoff depth decreased to 27.3 mm. Runoff regulation efficiency increased by 34.7%. Landscape fragmentation showed a significant positive correlation with runoff volume. Patch aggregation showed a significant positive correlation with runoff reduction rate. A dispersed green-space layout improved regulation efficiency by 18.6% compared with a centralized layout. These results indicate that the spatial structure of digital landscapes influences runoff regulation at the community scale. The GIS–InVEST coupling framework provides quantitative support for landscape optimization and spatial planning of sponge communities.

## Introduction

Rapid urbanization has continued to accelerate the expansion of impervious surfaces in high-density urban communities. Additionally, green-space patches have been progressively fragmented. These changes have reduced the capacity for surface runoff regulation and have further intensified urban waterlogging risk and hydrological imbalance [1]. Communities represent the fundamental unit of the urban hydrological system. The efficiency of runoff regulation at this scale directly influences the effectiveness of sponge city development. Landscape space serves as the physical carrier of runoff generation, concentration, and attenuation. Land-cover types, patch configurations, and spatial connectivity determine the processes of rainfall storage, infiltration, and transport. Consequently, the refined digital representation of landscape information forms the basis for the quantitative assessment of runoff regulation. Previous studies have largely relied on empirical formulas or single hydrological models. Under such approaches, the coupling relationship between landscape pattern evolution and runoff dynamics cannot be fully captured [2]. Although the Geographic Information System (GIS) has been applied to landscape data interpretation, the integration between GIS-based landscape analysis and ecosystem service models remains limited. As a result, the transformation pathway from digital landscape information to runoff regulation performance has not been clearly established. It is therefore necessary to develop a refined coupling analysis framework for high-density communities. Such a framework can identify the feedback relationship between landscape patterns and runoff regulation and support landscape optimization and design at the community scale.

Research on the relationship between urban landscapes and runoff regulation has expanded in several directions, including ecosystem services, hydrological simulation, green infrastructure configuration, and multi-scale regulation. Some studies have applied landscape pattern indices to analyze the influence of green spaces and impervious surfaces on runoff variation. However, most investigations have focused on the urban, watershed, or regional scale. Fine-scale spatial heterogeneity within communities has received limited attention. From the perspective of ecosystem service mechanisms, Vári et al. decomposed flood regulation services and systematically examined the contributions of vegetation cover, soil infiltration capacity, and hydrological connectivity to runoff regulation. Their results indicated that a single ecological factor could not explain differences in regulation efficiency. They therefore proposed a coordinated “structure–function–service” analytical framework, which provided a mechanistic reference for studying the relationship between landscape patterns and runoff regulation [3]. Focusing on runoff management at the urban parcel scale, Kändler et al. proposed a combined technical pathway of “street storage–distributed real-time control.” Validation with observed data showed that peak runoff was reduced by more than 30%. This finding demonstrated the engineering applicability of integrating spatial storage capacity with dynamic control strategies [4]. Pozdniakov et al. developed a physically based dual-channel digital filtering model. By introducing soil hydrodynamic parameters, the model improved the accuracy of groundwater runoff estimation and supported the separation of surface and subsurface runoff contributions [5]. Li et al. introduced a multi-level and multi-objective collaborative regulation concept into runoff management. Using machine learning prediction models, they established a closed-loop structure of “prediction–regulation–feedback,” which improved water-use efficiency while reducing runoff output [6]. Based on a flood regulation service supply–demand balance framework, Zhou and Wu developed a priority allocation model for green stormwater infrastructure. Their approach integrated service supply capacity with flood-risk demand and introduced a “supply–demand matching degree” indicator, which provided a quantitative basis for optimizing urban green-space allocation [7]. Schröder et al. applied the Integrated Valuation of Ecosystem Services and Trade-offs (InVEST) urban flood risk mitigation model to simulate heavy rainfall runoff and the effectiveness of nature-based solutions in Hannover, Germany. The study integrated surface runoff, social vulnerability, and infrastructure exposure into a unified analytical framework. The results showed that the InVEST model could support urban-scale flood risk identification and the prioritization of nature-based measures. However, its spatial units remain primarily at the urban sub-district level, and fine-scale landscape structures within communities are not fully represented [8]. Zhuang et al. developed a combined two-dimensional and three-dimensional evaluation method for environmental factors to assess the runoff reduction effects of urban green infrastructure. The study emphasized the coupling relationships among building morphology, green space distribution, and runoff pathways. The results indicated that the effectiveness of green infrastructure in runoff regulation was influenced not only by coverage area but also by spatial configuration and flow path organization. However, quantitative explanations of patch fragmentation, aggregation, and impervious surface connectivity at the community scale remain limited [9]. Kifayatullah et al. used high-resolution satellite imagery and GIS techniques to classify and evaluate urban green spaces. The study demonstrated that GIS provided fine-grained support for green infrastructure identification and spatial equity analysis. However, the study mainly focused on accessibility and ecological functions of green spaces and did not explicitly link these factors to runoff regulation processes [10]. Biag et al. employed GIS-based methods to identify urban stormwater and rainwater harvesting potential areas. The study highlighted the combined influence of topography, land cover, and drainage conditions in urban flood risk assessment. This study provides a reference for identifying spatial potential zones for stormwater regulation. However, it primarily focuses on urban-scale zoning and does not further examine how landscape patch organization within communities affects runoff reduction efficiency [11].

Wübbelmann et al. reported that extreme rainfall events projected for 2050 could weaken the ecosystem service capacity of urban flood regulation. However, nature-based solutions such as permeable pavements and bioretention systems could offset a significant portion of this loss. Their findings suggested that climate adaptation factors should be incorporated into runoff regulation design [12]. Longato et al. further proposed a three-dimensional evaluation system that integrated demand, cost–benefit analysis, and spatial suitability. This framework improved the allocation efficiency of community-level nature-based solutions [13]. By coupling dendroclimatology with hydrological modeling, Chen et al. revealed the indirect regulatory influence of the Pacific Decadal Oscillation on runoff variation. Their work suggested that local landscape analyses should be interpreted within a broader climatic context [14]. From the perspective of collaborative environmental governance, Hewitt found that specific improvement measures reduced runoff pollution while simultaneously mitigating leaching risks. This result highlighted the emerging linkage between runoff regulation and integrated ecological management [15]. Under special underlying surface conditions, Bhagwat et al. improved a snowmelt runoff model by introducing a regulation correction factor. The modification significantly enhanced simulation accuracy and provided methodological reference for runoff research in complex anthropogenic intervention scenarios [16]. In addition, some studies have attempted to integrate landscape indices such as fragmentation and aggregation with the InVEST model. However, parameter calibration often relied on indirect data, and validation with observed runoff data remained limited. As a result, the quantitative linkage between landscape indices and model outputs has not yet been fully established [17,18].

Based on existing studies, GIS, the InVEST model, green infrastructure assessment, and runoff scenario simulation have established a solid methodological foundation. However, three key aspects still require further development. First, most studies have been conducted at the urban, watershed, or district scale. Fine-scale heterogeneity within communities, such as short slope surfaces, fragmented green spaces, auxiliary impervious areas, and micro-topographic variations, has not been sufficiently represented. Second, although some studies can evaluate the overall regulatory effects of green space or nature-based solutions, the interpretation of spatial pattern metrics—such as patch fragmentation, aggregation, and impervious surface connectivity—remains limited. Third, while the InVEST model has strong applicability in stormwater regulation studies, its application at the community scale requires calibration with observed runoff data. Without such calibration, discrepancies may arise between model outputs and actual runoff responses.

In response to these limitations, this study focuses on runoff regulation assessment at the high-density urban community scale. Existing research has primarily examined the relationship between green infrastructure and runoff response at urban, watershed, or regional levels. The micro-scale landscape heterogeneity within communities, impervious surface connectivity, and green patch configuration have not been sufficiently quantified. In particular, when GIS-based spatial analysis and the InVEST model are directly downscaled from regional parameters to the community level, it becomes difficult to accurately capture runoff dynamics driven by short slope surfaces, fragmented green spaces, and localized impervious areas. This leads to a key research gap in this study: there is no continuous analytical chain linking digital landscape classification, runoff simulation, and landscape pattern interpretation at the community scale.

The significance of this study lies in integrating digital landscape identification, ecosystem service modeling, and observed runoff calibration into a unified framework, thereby providing a quantifiable, spatially explicit, and comparable methodological basis for sponge city community renewal. Unlike studies that focus only on green space area or impervious surface ratio, this work further examines how landscape spatial structure influences runoff regulation efficiency. It specifically emphasizes the roles of patch fragmentation, aggregation, impervious surface connectivity, and green space configuration in runoff generation and reduction processes at the community scale.

The objectives of this study are as follows:

(1) To develop an eight-class digital landscape classification system for community-scale runoff regulation analysis and clarify the hydrological functions of different surface types;
(2) To establish a coupled framework integrating GIS, the InVEST-Sediment Delivery Ratio (SDR) model, and landscape pattern metrics, and to calibrate and validate the model using observed runoff data;
(3) To compare runoff responses under current conditions and green infrastructure optimization scenarios, and to quantify the effects of increased green coverage, enhanced patch aggregation, and dispersed layout strategies on runoff reduction efficiency;
(4) To identify key spatial factors influencing community-scale runoff regulation and provide decision support for sponge-based retrofitting, green space allocation, and impervious surface connectivity control in high-density urban communities.

## Materials and methods

### Study area

The study area consisted of a typical high-density urban community located in the eastern region. The community is characterized by high building density. Residential buildings, road networks, paved surfaces, auxiliary hardened spaces, green patches, and small water bodies are interspersed within the area. This configuration creates a complex surface cover structure with a high proportion of impervious surfaces. The overall terrain is relatively flat, although short slopes and micro-topographic variations occur locally. After rainfall events, surface flow paths remain short, which leads to rapid runoff formation. Localized water accumulation risk is therefore relatively prominent. Climatic conditions show clear seasonal variation, with rainfall concentrated during the wet season. Short-duration rainfall events place continuous pressure on the community’s drainage and storage capacity.

The case area has three representative characteristics. First, high-density construction coexists with a large proportion of hardened surfaces, which reflects common structural features of stormwater regulation challenges in urban communities. Second, green-space patches are relatively small and spatially dispersed, which allows identification of how landscape spatial structure influences runoff processes. Third, the community boundary is clearly defined. This condition facilitates high-resolution digital landscape identification, monitoring point deployment, and scenario-based simulation analysis. The study area therefore provides an appropriate setting for evaluating runoff regulation effects and developing landscape optimization strategies at the community scale.

### Data sources

To improve the clarity and organization of the data description, the data sources were categorized into four groups: landscape identification data, meteorological data, observed runoff data, and spatial preprocessing data.

This study selected typical high-density urban communities in eastern China as the research area, and the data mainly used publicly available authoritative datasets, including the NLCD (2022 edition) published by the United States Geological Survey (USGS), long-term meteorological data from municipal meteorological stations in the research area, and runoff data observed by the research team in the field. NLCD data can be accessed through the USGS Earth Explorer platform (access link: https://earthexplorer.usgs.gov/). It has an original spatial resolution of 30 meters and containing 16 types of land cover. In this study, it was cropped and resampled to a resolution of 1 meter for cross validation with GIS interpreted landscape data. Meantime, the impermeable surface and green space data extracted by NLCD were input into the InVEST model, and compared with the runoff depth simulation results based on GIS interpreted data. In this study, the NLCD data were primarily used for cross-validation and result comparison. Digital landscape classification at the community scale was mainly based on the interpretation of high-resolution remote sensing imagery.

The evaluation of landscape classification relies on two core datasets: the 0.5m high-resolution remote sensing images obtained in 2022 (used for GIS interpretation to generate 8 types of landscape data), and the NLCD 2022 version of land cover data. The overall accuracy (OA = 92.3%) and Kappa coefficient (K = 0.89) were calculated through confusion matrix to verify the classification accuracy, ensuring that the landscape classification results meet the input requirements of the model.

Meteorological data is obtained through the official data sharing platform of the municipal meteorological department within a 20 km radius of the research area. The time resolution is monthly, the time range is from 2018 to 2023, and the time step is monthly. The data includes annual precipitation (1215 mm), monthly average precipitation (195 mm from June to September in the rainy season and 45 mm from December to February in the dry season), and annual average rainfall duration (1200 hours). They are used to calculate the rainfall erosivity factor (R) in the InVEST model and the potential runoff parameters in the runoff depth derivation formula, ensuring that the model reflects the long-term hydro meteorological conditions of the study area. The 42 sets of measured runoff data used for model calibration are rainfall event scale data collected by the research team at five monitoring points in the community from 2022 to 2023 (with a time step of the entire process of a single rainfall event), covering three scenarios: light rain, moderate rain, and heavy rain. It should be noted that the land use data in this study is static data (based on 2022 remote sensing images and NLCD 2022 version), and does not involve multi-period land use changes. The analysis of temporal changes is limited to climate factors (monthly and inter-annual fluctuations in rainfall), and does not include the temporal dynamic evolution of land use. Five monitoring points were installed along major hardened flow paths, green-space transition zones, and local low-lying units. This configuration ensured coverage of different surface types and runoff response locations. The monitoring layout captured runoff variation under the spatial heterogeneity of the community and provided a data basis for parameter calibration and model validation.

All datasets were spatially coupled through GIS, and a unified CGCS2000 coordinate system and 1m × 1m grid resolution were used to construct an integrated database of “terrain landscape soil meteorology observation.”

### Digital landscape classification and land-cover definition

The term *digital landscape* in this study does not directly adopt the original categories from general land-cover databases. Instead, it refers to a functional spatial representation system developed for community-scale runoff regulation analysis. The system was established through the interpretation of high-resolution remote sensing imagery, terrain information, and surface-cover characteristics. Conventional land-cover classifications mainly serve regional-scale surface type identification. Their category boundaries are relatively coarse and cannot adequately capture micro-scale paved structures, green patch variation, or auxiliary hardened spaces within communities. In contrast, digital landscape classification emphasizes differences in the hydrological roles of surface units during runoff generation, storage, infiltration, and concentration. It therefore considers not only cover attributes but also spatial organization and hydrological response characteristics.

Based on the internal surface structure of the community, eight categories of digital landscape units were defined: building-covered space, roads and linear paved space, plazas and auxiliary hardened areas, tree-covered green space, shrub green space, grassland, water bodies, and bare soil. Building-covered space refers to residential and public-service buildings together with the permanent roof projection area. Roads and linear paved space include vehicular lanes, pedestrian paths, and other continuous linear hardened corridors. Plazas and auxiliary hardened areas include paved activity spaces, parking areas, and hardened surfaces surrounding buildings. Tree-covered green space, shrub green space, and grassland distinguish vegetation layers that differ in rainfall interception, root-mediated water conduction, and surface infiltration capacity. Water bodies include landscape water surfaces and stable water storage units. Bare soil represents temporarily exposed ground without pavement or stable vegetation cover.

Compared with the NLCD, this classification system differs in two main aspects. First, it operates at a finer spatial scale and can identify small hardened units and transitional green spaces within the community. Second, the classification criteria are closely aligned with runoff regulation analysis. This structure provides more suitable inputs for impervious surface connectivity analysis, green-patch aggregation measurement, and scenario-based optimization. NLCD data were used only for cross-validation and simulation result comparison and did not replace the community-scale digital landscape classification.

Green-space types were defined using a hierarchical structure. Tree-covered green space corresponds to vegetation units with clear canopy coverage and well-developed root systems. Shrub green space refers to mid- and low-layer woody vegetation units. Grassland represents areas dominated by herbaceous surface vegetation. These three categories differ significantly in rainfall interception capacity, root water conduction, surface roughness, and soil disturbance. They were therefore treated separately in model parameterization and landscape pattern analysis rather than being merged into a single green-space category.

The definitions of the eight digital landscape units are presented in Table 1.

**Table 1:** Definition of digital landscape units.

Category No.	Digital Landscape Category	Spatial Definition	Hydrological Functional Characteristics
**1**	Building-covered space	Roof surfaces and permanent structural coverage	Highly impervious, rapid runoff generation
**2**	Roads and linear paved space	Roads, pedestrian paths, and other linear hardened surfaces	Strong connectivity, clear runoff pathways
**3**	Plazas and auxiliary hardened areas	Plazas, parking areas, and auxiliary paved surfaces	Rapid runoff generation with local flow accumulation
**4**	Tree-covered green space	Green areas with distinct tree canopy coverage	Strong interception and enhanced infiltration
**5**	Shrub green space	Areas dominated by mid- and low-layer woody vegetation	Effective interception and runoff attenuation
**6**	Grassland	Herbaceous vegetation coverage	Surface storage and gradual runoff release
**7**	Water bodies	Stable landscape water surfaces	Water storage and regulation units
**8**	Bare soil	Temporarily exposed ground without pavement	Sensitive to rainfall erosion and rapid hydrological response

### Model framework construction and core algorithm design

This study adopted the SDR module of the InVEST model. The selection was based on the research objective. The study aimed to evaluate the relative effects of landscape structure on surface runoff regulation at the community scale rather than to identify urban flood inundation risk areas. The Urban Flood Risk Mitigation module is more suitable for analyses related to stormwater inundation risk, exposed flooded units, and disaster mitigation demand. Its outputs primarily emphasize flood-risk reduction capacity. In contrast, this study focused on the combined influence of landscape patch type, spatial configuration, slope conditions, and surface cover differences on runoff generation, storage, and transport. Therefore, a process-based module capable of integrating terrain factors, land-cover characteristics, soil attributes, and spatial structural information was required.

The SDR module was originally developed to represent surface material transport and retention processes. However, its parameter system effectively reflects the combined influence of slope gradient, slope length, surface cover conditions, soil erodibility, and surface protection factors on surface flow accumulation and transport capacity. At the community scale, these factors are closely associated with runoff generation processes. In this study, SDR outputs were integrated with infiltration correction, potential runoff derivation, and landscape pattern indices to construct an indirect quantification pathway for runoff regulation effects. This approach does not equate sediment transport with runoff processes. Instead, it uses the module’s ability to characterize spatial resistance, transport sensitivity, and slope-response dynamics to represent differences in runoff regulation under varying community landscape structures.

To reduce potential bias caused by a single proxy variable, observed runoff data were further introduced for parameter calibration. Landscape pattern indices were also analyzed together with simulation outputs. This strategy enhanced both the applicability of the model at the community scale and the interpretability of the results.

The evaluation framework and scenario simulation in this study serve different functions. The evaluation framework is used for community-scale baseline identification, parameter calibration, runoff regulation quantification, and extraction of key spatial factors, forming the foundational structure of the entire analysis. The scenario simulation is built upon this framework and is used to compare runoff response differences under different optimization strategies by adjusting green space coverage, patch aggregation, and spatial configuration. Therefore, the former is designed for baseline assessment and mechanism identification, while the latter focuses on optimization evaluation and pathway comparison.

The runoff regulation model developed in this study is based on GIS spatial analysis for data processing and integrates the ecological process simulation functionality of the InVEST-SDR module. Using 22 core formulas, the model implements a complete logical chain from landscape data interpretation to runoff regulation efficiency quantification [19]. The model input layer uses a 1 m resolution Digital Elevation Model (DEM) and 0.5 m high-resolution remote sensing imagery. GIS is employed to extract topographic parameters and classify landscape types. Slope (α, in radians) is calculated based on the elevation change rate within a 3 × 3 grid window using the vector composition method:


$$\begin{array}{c} \alpha =\text{arctan}(\sqrt{{\left(\frac{z_{i+\text{1},j}-z_{i,j}}{\text{cellsize}}\right)}^{\text{2}}+{\left(\frac{z_{i,j+\text{1}}-z_{i,j}}{\text{cellsize}}\right)}^{\text{2}}}) \end{array}$$ (1)

where $z_{i+\text{1},j}$ and $z_{i,j+\text{1}}$ represent the elevations of neighboring grid cells in the *x* and *y* directions for thse target cell (i,j), and cellsize=1m [20]. To meet the InVEST model requirements for topographic input, slope in radians is converted to degrees:


$$\begin{array}{c} \theta =\alpha \times \frac{\text{1}\text{8}\text{0}}{\pi } \end{array}$$ (2)

Slope length (L) is calculated using a modified algorithm suitable for microtopography at the community scale, incorporating flow accumulation (A, in m^2^) for quantification:


$$\begin{array}{c} L={\left(\frac{\text{2}\text{2}.\text{1}\text{3}\times (A+\text{2}\text{2}.\text{1}\text{3})}{\text{1}\text{0}\text{0}\text{0}}\right)}^{\text{0}.\text{5}} \end{array}$$ (3)

The formula introduces a 22.13 m^2^ flow accumulation threshold to correct biases in short slope regions (<50 m), matching the actual terrain characteristics of the study area [21].

After landscape type classification, accuracy is validated using a confusion matrix. Overall Accuracy (OA) is calculated as:


$$\begin{array}{c} OA=\frac{\sum_{i=\text{1}}^{n}x_{ii}}{\sum_{i=\text{1}}^{n}\sum_{j=\text{1}}^{n}x_{ij}} \end{array}$$ (4)

The Kappa coefficient (K) is:


$$\begin{array}{c} K=\frac{N\sum_{i=\text{1}}^{n}x_{ii}-\sum_{i=\text{1}}^{n}(x_{i+}\times x_{+i})}{N^{\text{2}}-\sum_{i=\text{1}}^{n}(x_{i+}\times x_{+i})} \end{array}$$ (5)

where n = 8 is the number of landscape types, $x_{ii}$ is the diagonal element of the confusion matrix (correctly classified pixels), N is the total number of pixels, and $x_{i+}$ and $x_{+i}$ are the actual and classified total pixel counts of type i. OA and Kappa were used to assess whether the landscape classification satisfied the accuracy requirements for model input [22]. The InVEST-SDR module quantifies the ecosystem’s sediment retention capacity using the Universal Soil Loss Equation (USLE) factors, indirectly representing runoff regulation efficiency [23]. The rainfall erosivity factor (R) considers the seasonality of the study area, with 70% of precipitation occurring from June to September. Monthly R values are calculated as:


$$\begin{array}{c} R_{\text{month}}=\text{0}.\text{2}\text{0}\text{8}\times \overset{\text{1}.\text{7}\text{2}\text{6}}{\underset{\text{month}}{P}} \end{array}$$ (6)

P stands for the monthly total precipitation in the study area (unit: mm). Annual R values is shown in Equation (7):


$$\begin{array}{c} R_{\text{year}}=\sum_{\text{month}=\text{1}}^{\text{1}\text{2}}R_{\text{month}} \end{array}$$ (7)

where $R_{\text{month}}$ is the monthly precipitation (mm). Monthly precipitation data from 2018 to 2023 were used to calculate seasonal R values for the rainy and dry periods [24].

Soil erodibility factor K is determined from soil particle composition (sand S, silt Si, clay Cl, in %) and organic matter (OM, in %):


$$\begin{array}{c} K_{\text{base}}=\text{0}.\text{0}\text{0}\text{3}\text{2}+\text{0}.\text{2}\text{2}\text{9}\times e^{-\text{0}.\text{0}\text{2}\text{5}\text{1}\times S}+\text{0}.\text{0}\text{0}\text{0}\text{3}\times Si-\text{0}.\text{0}\text{0}\text{2}\text{5}\times OM \end{array}$$ (8)

Clay (clay content, unit:%) is a key indicator that characterizes the composition of soil particles, and together with sand particles (S, sand) and silt particles (Si, silt), determines the soil texture characteristics. The final K value is obtained from the organic matter correction calculation:


$$\begin{array}{c} K=K_{\text{base}}\times (\text{1}-\text{0}.\text{2}\text{5}\times \frac{OM}{OM+\text{2}.\text{5}\text{9}}) \end{array}$$ (9)

Slope-length LS factor is calculated in segments:

For θ < 9%:


$$\begin{array}{c} LS={\left(\frac{L}{\text{2}\text{2}.\text{1}\text{3}}\right)}^{\text{0}.\text{5}}\times (\text{0}.\text{0}\text{6}\text{5}+\text{0}.\text{0}\text{4}\text{5}\times \theta +\text{0}.\text{0}\text{0}\text{2}\text{5}\times \theta^{\text{2}}) \end{array}$$ (10)

For θ ≥ 9%:


$$\begin{array}{c} LS={\left(\frac{L}{\text{2}\text{2}.\text{1}\text{3}}\right)}^{\text{0}.\text{5}}\times (\text{0}.\text{1}\text{3}\text{8}+\text{0}.\text{1}\text{5}\text{3}\times \theta +\text{0}.\text{0}\text{0}\text{3}\text{3}\times \theta^{\text{2}}) \end{array}$$ (11)

The setting criteria for the segmentation threshold (θ = 9%) and coefficients (0.065, 0.045, 0.0025; 0.138, 0.153, 0.0033) are as follows:

(1) Segmented threshold (θ = 9%): Referring to the “Soil Erosion Classification and Grading Standards” and urban micro terrain characteristics, combined with the statistical results of community slope in the study area (80% of the area slope < 9%), the slope is divided into two categories: gentle slope (< 9%) and steep slope (≥ 9%), mainly adapted to the terrain characteristics of community short slope (< 50 m);
(2) Coefficient values: A community scale correction scheme based on the general soil erosion equation [25], referring to the parameter calibration experience of similar high-density urban community runoff simulation studies, the coefficient was adjusted to compensate for the amplification effect of runoff acceleration on erosion in steep slope areas. Finally, 42 sets of measured runoff data were verified to ensure that the LS factor calculation was consistent with the actual erosion law in the region. The segmentation accounts for the amplification effect of steep slopes on erosion. Vegetation cover factor C is linked to NDVI and fractional vegetation cover $f_{c}$:

$$\begin{array}{c} NDVI=\frac{\rho_{\text{NIR}}-\rho_{\text{Red}}}{\rho_{\text{NIR}}+\rho_{\text{Red}}} \end{array}$$ (12)

where $\rho_{\text{NIR}}$ and $\rho_{\text{Red}}$ represent the reflectance values of the near-infrared and red bands, respectively. Fractional vegetation cover ($f_{c}$) is calculated as:


$$\begin{array}{c} f_{c}=\frac{NDVI-NDVI_{\text{min}}}{NDVI_{\text{max}}-NDVI_{\text{min}}} \end{array}$$ (13)

where $NDVI_{\text{min}}$ = 0.05 for bare soil, and $NDVI_{\text{max}}$ = 0.85 for dense vegetation. The final vegetation cover factor (C) is computed as:


$$\begin{array}{c} C=\text{0}.\text{6}\text{5}-\text{0}.\text{5}\text{8}\times f_{c} \end{array}$$ (14)

The soil conservation factor (P) is determined based on the efficiency of implemented measures (η):


$$\begin{array}{c} P=\text{1}-\text{0}.\text{7}\times \eta \end{array}$$ (15)

where η = 0.6 for grassed swales, η = 0.8 for permeable pavement, and η = 0 when no measure is applied, quantifying the erosion reduction effectiveness of different engineering interventions [26].

Potential soil loss is calculated using the USLE factors:


$$\begin{array}{c} A=R_{\text{year}}\times K\times LS\times C\times P \end{array}$$ (16)

Next, the sediment delivery ratio ($SDR_{x}$) for each grid cell *x* is computed using the core InVEST-SDR formula:


$$\begin{array}{c} SDR_{x}=\frac{\text{1}}{\text{1}+\text{2}\text{1}.\text{9}\text{8}\times (LS_{x}\times C_{x}\times P_{x}{)}^{-\text{0}.\text{8}}} \end{array}$$ (17)

Runoff depth ($R_{x}$) is derived by coupling potential runoff with actual infiltration. The potential runoff for cell *x* is:


$$\begin{array}{c} P_{\text{pro},x}=P_{\text{ann}}\times (\text{1}-\text{0}.\text{3}\times f_{\text{green},x}) \end{array}$$ (18)

where $P_{\text{ann}}$ = 1215 mm represents the annual average precipitation, and $f_{\text{green},x}$ is the proportion of green space in grid cell *x*. The base soil infiltration rate $f_{\text{base},x}$ is related to the impervious surface fraction ($f_{\text{imper},x}$):


$$\begin{array}{c} f_{\text{base},x}=\text{2}.\text{5}-\text{2}.\text{0}\times f_{\text{imper},x} \end{array}$$ (19)

The actual infiltration rate, adjusted for vegetation root effects, is:


$$\begin{array}{c} f_{\text{act},x}=f_{\text{base},x}\times (\text{1}+\text{0}.\text{4}\times f_{c}) \end{array}$$ (20)

The annual actual infiltration volume is then:


$$\begin{array}{c} F_{x}=f_{\text{act},x}\times T_{\text{rain}} \end{array}$$ (21)

where $T_{\text{rain}}$ = 1200 h represents the annual rainfall duration. Finally, runoff depth is calculated as:


$$\begin{array}{c} R_{x}=\text{0}.\text{7}\text{2}\times (P_{\text{pro},x}-\frac{F_{x}}{\text{1}\text{0}})\times SDR_{x}+\text{1}.\text{3}\text{5} \end{array}$$ (22)

where 0.72 is the correlation coefficient between SDR and runoff, and 1.35 is a base runoff adjustment factor, both calibrated using measured data.

### Model parameter calibration and statistical analysis

Model parameter calibration was based on 42 sets of measured data collected from five monitoring points within the community during 2022–2023, covering light rain (5–15 mm), moderate rain (15–30 mm), and heavy rain (>30 mm). Sensitivity analysis was conducted to identify key parameters, with the sensitivity coefficient calculated as:


$$\begin{array}{c} S=\frac{\Delta R/R}{\Delta \alpha /\alpha } \end{array}$$ (23)

where ΔR/R represents the relative change in simulated runoff depth, and Δα/α =5% is the relative change in the parameter. Model accuracy was evaluated using the coefficient of determination ($R^{\text{2}}$) and mean relative error (MRE):


$$\begin{array}{c} R^{\text{2}}=\text{1}-\frac{\sum_{i=\text{1}}^{m}(R_{\text{obs},i}-R_{\text{sim},i}{)}^{\text{2}}}{\sum_{i=\text{1}}^{m}(R_{\text{obs},i}-\overset{―}{R_{\text{obs}}}{)}^{\text{2}}} \end{array}$$ (24)

$$\begin{array}{c} MRE=\frac{\text{1}}{m}\sum_{i=\text{1}}^{m}\frac{\left|R_{\text{sim},i}-R_{\text{obs},i}\right|}{R_{\text{obs},i}}\times \text{1}\text{0}\text{0} \end{array}$$ (25)

where m = 42 is the number of data sets, $R_{\text{obs},i}$ and $R_{\text{sim},i}$ are the observed and simulated runoff depths, respectively, and $\overset{―}{R_{\text{obs}}}$ is the mean observed runoff depth [27]. The association between landscape pattern and runoff regulation effectiveness was quantified using Pearson correlation analysis, with the correlation coefficient calculated as:


$$\begin{array}{c} =\frac{\sum_{i=\text{1}}^{k}(X_{i}-\overset{―}{X})\times (Y_{i}-\overset{―}{Y})}{\sqrt{\sum_{i=\text{1}}^{k}(X_{i}-\overset{―}{X}{)}^{\text{2}}}\times \sqrt{\sum_{i=\text{1}}^{k}(Y_{i}-\overset{―}{Y}{)}^{\text{2}}}} \end{array}$$ (26)

where k = 125 represents the number of community grid cells, $X_{i}$ is the landscape pattern index, $Y_{i}$ is the corresponding grid cell runoff depth ($R_{x}$), and $\overset{―}{X}$ and $\overset{―}{Y}$ are the mean values of the variables [28,29]. Statistical significance was tested using the t-statistic:


$$\begin{array}{c} t=|r|\times \sqrt{\frac{k-\text{2}}{\text{1}-r^{\text{2}}}} \end{array}$$ (27)

The statistical analysis comprised four parts. First, sensitivity analysis was conducted to identify key parameters, including vegetation cover factor (C), slope-length and slope-gradient factor (LS), and infiltration rate, which then guided parameter calibration. Second, model fit was evaluated using the coefficient of determination (R^2^) and mean relative error, based on 42 rainfall events measured at the community scale between 2022 and 2023. Third, Pearson correlation analysis was used to examine linear relationships between landscape fragmentation, patch aggregation, and runoff response indicators, with significance levels assessed via t-tests [30,31]. Fourth, the regulatory effect of landscape optimization scenarios was assessed by calculating the differences and relative changes in runoff depth before and after optimization.

## Results

The results are presented in three parts: model validation and parameter calibration, current runoff regulation performance, and landscape pattern responses under optimization scenarios.

### Model validation and parameter calibration results

Comparison of runoff simulation results based on the NLCD and GIS-interpreted data showed an average runoff depth deviation of 2.1 mm, indicating good consistency between the two sources for community-scale landscape identification. Cross-validation results confirmed that the digital landscape classification derived from high-resolution remote sensing imagery was suitable for subsequent model inputs and scenario simulations.

Sensitivity analysis revealed that the vegetation C, LS, and infiltration rate were highly influential, with sensitivity coefficients of 0.82, 0.65, and 0.43, respectively. These factors were identified as key variables for parameter calibration. During calibration, the C factor for tree-covered green space was adjusted from 0.06 to 0.05, reducing the relative error at the corresponding monitoring points from 8.7% to 1.9%. In areas with slopes exceeding 8%, the LS factor was adjusted from 0.43 to 0.48, which more accurately reflected runoff acceleration under steep-slope conditions. After calibration, the model achieved a R^2^ of 0.89 and an average relative error of 1.3%, demonstrating high fitting accuracy at the community scale.

**Fig 1:**
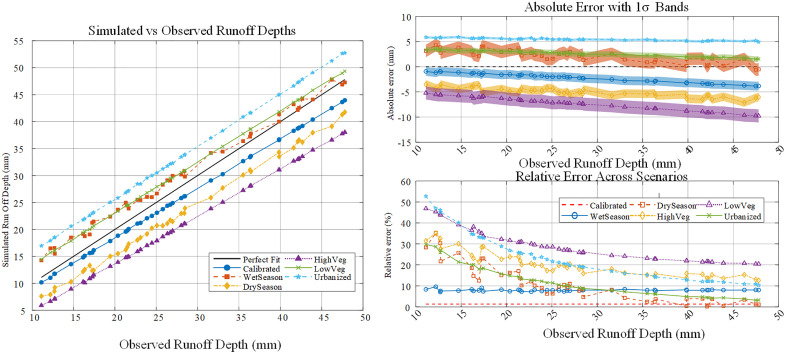
Performance evaluation of the GIS–InVEST coupled model.

Fig 1 presents the model calibration results. Simulated runoff depths under different typical scenarios closely matched observed values, with most samples ranging from 10 to 50 mm. Absolute errors were narrow, and relative errors remained generally low, indicating that the model maintains good stability and applicability across varying rainfall conditions.

Fig 1 shows that the simulated runoff depths under six representative scenarios are in strong agreement with the observed values, with most samples concentrated in the 10–50 mm range. The absolute error remains within a narrow interval, and the relative error is generally low. These results indicate that the model errors are not randomly distributed but are primarily concentrated in spatial units with rapid slope variation and pronounced differences in vegetation coverage. The C-factor and LS-factor exhibit higher sensitivity, suggesting that runoff processes at the community scale are more strongly influenced by vegetation interception capacity and short-slope flow accumulation conditions. In contrast, although infiltration rate is also an important factor, its marginal regulatory effect is weaker under high impervious surface conditions, where land cover type and slope structure differences play a more dominant role.

### Current runoff regulation results

**Fig 2:**
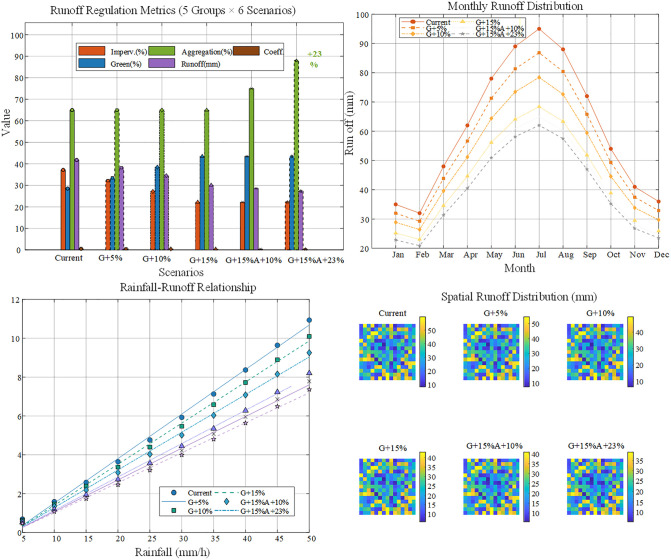
Current runoff regulation effectiveness assessment.

Fig 2 presents the assessment results of runoff regulation performance under current conditions.

Fig 2 shows that under the current landscape configuration, the proportion of impervious surfaces in the community is 37.2%, with an annual mean runoff depth of 41.8 mm and a runoff coefficient of 0.52. These results indicate a relatively high level of surface runoff generation under the existing spatial configuration, suggesting that the community still has considerable potential for improving stormwater retention and regulation capacity. The current results reveal two key characteristics. First, the relatively high proportion of impervious surfaces enables rapid conversion of rainfall into surface runoff, significantly limiting opportunities for infiltration and rainwater detention. Second, the regulatory effect of green spaces is largely confined to localized areas and has not yet formed a continuous buffering structure. As a result, the overall spatial organization efficiency remains low.

### Optimization scenario and landscape pattern response results

**Fig 3:**
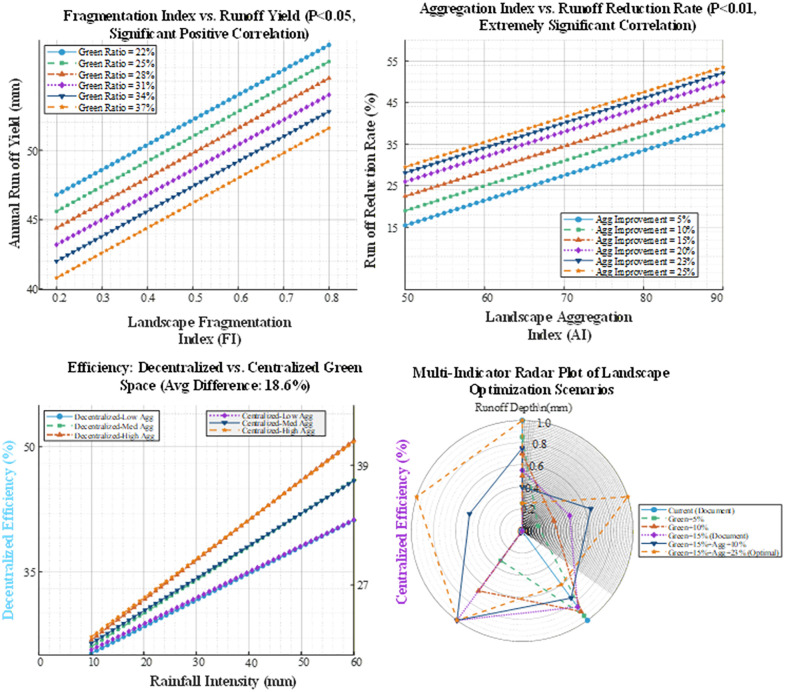
Optimized scenario runoff regulation assessment results.

To evaluate the regulatory effects of landscape optimization, an optimized scenario was established in which green-space coverage increased by 15% and patch aggregation increased by 23%. Under this scenario, the community’s mean annual runoff depth decreased from 41.8 mm to 27.3 mm, corresponding to a 34.7% improvement in runoff regulation efficiency. Fig 3 presents the results of the optimized scenario assessment.

Fig 3 shows a significant positive correlation between landscape fragmentation and runoff volume, as well as a strong positive correlation between patch aggregation and runoff reduction rate. Comparative results of different configuration strategies indicate that a dispersed green space layout achieves 18.6% higher regulation efficiency than a concentrated layout. The observed improvements under optimized scenarios are not solely driven by an increase in total green space. The increase in green coverage expands the spatial capacity for rainfall interception and surface water storage. As patch aggregation increases, previously isolated green units gradually form more continuous ecological buffering structures, which extend surface flow paths and reduce runoff concentration speed. Meanwhile, increased connectivity of impervious surfaces strengthens rapid runoff pathways, thereby amplifying runoff generation effects. The baseline results are used to identify the current runoff regulation status of the community, while the optimized scenario results are used to compare improvement magnitudes under different landscape adjustment strategies.

## Discussion

### High consistency in landscape classification

The small average runoff depth deviation of 2.1 mm between NLCD data and GIS-interpreted data indicates that community-scale landscape identification is largely consistent across different data sources. This consistency does not imply that the two datasets are identical, but rather that they accurately capture key regulatory units, particularly impervious surfaces, green spaces, and water bodies. The areas most sensitive to runoff regulation are those with significant differences in surface infiltration capacity. As long as the spatial delineation between hardened surfaces and vegetated areas remains stable, model outputs show minimal deviation. High-resolution remote sensing imagery further enhances the identification of micro-scale surface features, such as road edges, auxiliary paved areas, and scattered green patches, which are incorporated into the raster representation. This high-precision spatial data underpins the strong agreement observed in cross-validation and highlights the need for accurate landscape classification in community-scale runoff assessment.

### Mechanisms underlying key parameter sensitivity differences

The high sensitivity of the *C* factor (vegetation cover), *LS* factor (slope length–gradient), and infiltration rate reflects that community runoff processes are primarily controlled by surface cover, micro-topography, and infiltration capacity. The *C* factor shows the highest sensitivity due to its direct relation to green-space coverage. Vegetation alters surface roughness, rainfall interception, root-mediated water conduction, and topsoil structure, thereby exerting strong front-end regulation on runoff formation. The *LS* factor ranks second in sensitivity, indicating that even within short slopes and micro-topography typical of communities, the coupled effect of slope length and gradient accelerates surface flow. When slope increases, surface water residence time decreases, local infiltration opportunities diminish, and runoff depth rises sharply. Although infiltration rate also significantly influences runoff, its sensitivity is slightly lower. In high-density communities with extensive impervious surfaces, the potential impact of increased infiltration is limited; thus, its marginal regulatory effect is weaker than that of vegetation cover and slope-driven flow. The substantial reduction in errors following parameter calibration indicates that the original parameter system was reasonable, with residual errors concentrated in specific surface types and steep-slope areas rather than reflecting instability in the overall model structure.

### Intrinsic reasons for high runoff under current conditions

Under existing conditions, both mean annual runoff depth and runoff coefficient are relatively high. This is not solely due to insufficient green space, but rather to a structural mismatch between community landscape configuration and surface hydrological processes. High proportions of impervious surfaces reduce storage and infiltration capacity, causing rainfall to convert rapidly into surface runoff. Meanwhile, green patches exist but are small and scattered, preventing the formation of continuous buffers or interception zones. Spatially, stronger connectivity among hardened surfaces creates direct runoff pathways, placing greater runoff concentration pressure on local low-lying areas. In other words, the community does not lack regulatory units, but the connectivity between these units is insufficient to support synergistic runoff reduction. Individual green patches still provide ecological function, but when separated by roads, auxiliary paved surfaces, and scattered hardened areas, the overall retention capacity is significantly reduced. This explains why community-scale studies cannot rely solely on coverage proportion but must also consider landscape structural connectivity and patch configuration.

### Mechanisms behind the significant improvement under the optimized scenario

The increase in runoff regulation efficiency under the optimized scenario is driven not only by the expansion of green-space area but also by the synergistic effects of spatial configuration. As green-space coverage rises, the capacity for rainfall interception, surface storage, and soil infiltration expands, initially reducing total runoff. When patch aggregation also increases, previously scattered small green patches form more continuous ecological blocks. This extends the spatial pathways for rainfall movement, slows surface flow, and allows local runoff reduction effects to propagate over a larger area [32]. In other words, increasing green-space area addresses whether regulatory space exists, while higher aggregation determines whether these spaces can interact to form functional networks. When both factors improve together, internal retention chains and buffer structures within the community become more complete, producing a marked enhancement in runoff reduction. These findings indicate that sponge city upgrades should not rely solely on expanding green space; spatial organization is equally crucial for achieving effective regulation. These findings are consistent with the conclusions of Study [7], which reported that the spatial configuration of green stormwater infrastructure significantly affects the supply–demand matching of flood regulation services. They also align with Study [13], which emphasized that the efficiency of nature-based solutions is constrained by spatial adaptability. However, this study further refines the analysis by downscaling it to the community unit level and quantitatively evaluating the combined effects of increased green coverage and enhanced patch aggregation on runoff reduction magnitude.

### Reasons for differences in fragmentation, aggregation, and configuration patterns

Higher landscape fragmentation leads to greater runoff generation because green and permeable areas become segmented, disrupting continuous infiltration interfaces. In contrast, higher green space aggregation enables the formation of more integrated retention surfaces and transitional zones, thereby enhancing runoff reduction capacity. The superior performance of a dispersed green space layout does not contradict the benefits of an aggregated layout, as the two operate at different spatial levels. Aggregated configurations improve local structural integrity, while dispersed layouts expand interception coverage across multiple runoff initiation points. In high-density communities, broad spatial coverage is crucial for preventing rapid hydrological connectivity and runoff concentration. The findings of this study—namely, that increased landscape fragmentation intensifies runoff output and that higher aggregation enhances runoff reduction capacity—are consistent with the conclusions of Study [9], which emphasized that the effectiveness of green infrastructure in runoff regulation is strongly governed by spatial form. Compared with existing studies, this work further demonstrates that dispersed layouts achieve higher overall regulation efficiency within high-density community contexts. This difference suggests that runoff initiation points in communities are more spatially dispersed, and that single centralized configurations are insufficient to provide system-wide interception advantages.

### Reasons why impervious surface connectivity becomes a key driver

Runoff increases significantly as impervious surface connectivity rises, reflecting a clear hydrological mechanism. Once hardened surfaces form continuous channels, rainfall rapidly converges along paths of least resistance, compressing surface storage and infiltration processes. In high-density communities, roads, plazas, and hardened zones surrounding buildings often form such fast-flow networks. Compared with total impervious surface proportion, connectivity better explains why some communities exhibit different runoff responses despite similar levels of surface hardening. The proportion indicates how much impervious surface exists, whereas connectivity determines how efficiently water is transmitted. In high-density urban areas, connectivity is often more directly linked to sudden runoff surges than surface coverage alone. Therefore, reducing impervious area is not sufficient; breaking continuous hardened chains, introducing permeable transition zones, and adding micro-buffer units are necessary to structurally mitigate rapid runoff amplification. This interpretation is consistent with Study [3], which identified hydrological connectivity as a central factor in flood regulation services. It also aligns with Study [32], which demonstrated that the source–sink relationship of urban green infrastructure–runoff systems is strongly influenced by spatial structure. The contribution of this study lies in explicitly identifying impervious surface connectivity as a key triggering factor for rapid runoff amplification at the community scale, and incorporating it into the landscape optimization analysis framework.

### Implications for community landscape optimization

Runoff regulation at the community scale is not determined by a single indicator but is jointly shaped by landscape composition, spatial configuration, and micro-topography. High-resolution digital landscape representation improves the identification of spatial heterogeneity, while parameter calibration enhances the reliability of runoff simulation. The results indicate that regulation efficiency depends on whether spatial structure can delay surface runoff, enhance infiltration, and weaken the rapid connectivity of impervious surfaces. This shifts the analytical focus from simple area-based comparisons to structural coordination and provides a basis for subsequent planning recommendations.

## Limitations and future aspects

This study has three main limitations. First, in terms of data, it is based on 2022 high-resolution remote sensing imagery, NLCD reference data, meteorological records from 2018 to 2023, and 42 sets of observed runoff data. These datasets support current-state identification and scenario comparison at the community scale but do not form a continuous multi-temporal dataset of landscape evolution. Therefore, the analysis is more suitable for explaining the correspondence between current spatial patterns and runoff regulation rather than long-term landscape dynamics. Second, at the model level, the study couples the InVEST-SDR model with runoff derivation equations to represent surface regulation processes at the community scale. This framework captures the integrated effects of slope, land cover, soil properties, and spatial configuration on runoff generation, retention, and transport. However, subsurface drainage systems, soil microbial processes, pipe-network backflow, and short-duration extreme rainfall events are not explicitly included. As a result, the model is better suited for relative comparisons rather than high-resolution engineering simulation of urban drainage systems. Third, in terms of generalizability, the study focuses only on typical high-density urban communities characterized by clear boundaries, high impervious surface ratios, and fragmented green space patterns. The findings are highly applicable to aging residential districts, compact public-service communities, and high-density mixed-use areas. However, their direct applicability to low-density residential areas, mountainous communities, or large-scale urban districts still requires further validation.

Future research should proceed along three directions. First, incorporating multi-temporal landscape data and longer time-series rainfall–runoff observations to improve dynamic interpretation of landscape–hydrology relationships. Second, integrating subsurface drainage networks, soil biophysical processes, and extreme weather events to enhance model realism in complex urban hydrological systems. Third, expanding the range of study sites to enable cross-city and cross-form comparative analysis, thereby improving model applicability and scenario generalization capability.

## Policy recommendations

Optimization of community-scale runoff regulation should not rely solely on increasing green space area, but instead shift toward coordinated configuration of spatial structure and regulation nodes. Based on the results of this study:

First, priority should be given to reducing continuous impervious surface chains. Road edges, paved squares, building peripheries, parking areas, and auxiliary hardened zones often form rapid runoff pathways. These can be mitigated through permeable pavement replacement, insertion of micro-green spaces, construction of sunken boundary strips, and installation of transitional buffer units, thereby weakening impervious connectivity and reducing rapid runoff amplification.

Second, green space configuration should be optimized rather than simply increasing total green area. In high-density communities, green spaces should avoid excessive concentration in a few landscape nodes. Instead, distributed regulation units should be placed at key runoff initiation points and flow pathways, while localized aggregation should be maintained to form effective ecological buffering zones. This strategy increases spatial coverage while enhancing local retention and reduction capacity.

Third, digital landscape identification results should be integrated into sponge city renewal and spatial planning processes. Community renewal, road reconstruction, auxiliary space upgrading, and green infrastructure redesign should jointly incorporate landscape pattern metrics, impervious surface connectivity indicators, and runoff regulation performance indicators to support quantitative and comparable decision-making. Priority should be given to units with highly connected impervious surfaces, fragmented green spaces, and localized low-lying accumulation risks, as these areas offer the greatest potential for improving community-scale stormwater regulation capacity.

From a planning governance perspective, the results of this study indicate that effective community-scale stormwater management depends not only on increasing green coverage but also on improving the spatial relationship between green and impervious systems. Fine-grained spatial optimization in high-density residential areas is more effective than relying solely on macro-level greening indicators. This perspective provides actionable guidance for community renewal, brownfield redevelopment, and resilience improvement in urban drainage systems under the sponge city framework.

## Conclusions

This study integrated GIS with the InVEST-SDR model to develop a community-scale runoff regulation assessment framework, which was applied to a representative high-density urban community. The results show that digital landscape classification provides reliable input data, and the calibrated framework achieves satisfactory accuracy at the community scale. Under current conditions, the community exhibits relatively high runoff output, while increasing green space coverage and patch aggregation significantly improves runoff regulation efficiency. The analysis indicates that landscape fragmentation contributes to increased runoff generation, whereas higher patch aggregation enhances runoff reduction capacity. Impervious surface connectivity is identified as a key trigger of rapid runoff formation. These findings suggest that community-scale runoff regulation is not only dependent on green space area but is also strongly influenced by spatial configuration and the connectivity of impervious surfaces. The proposed framework provides quantitative support for digital landscape optimization and fine-grained stormwater management in sponge-like urban communities.

## Supporting information

S1 Data The relevant data in the article can be viewed in the supporting information “Dataset. zip.” (ZIP)
